# Evidence requirements of permanently listed digital health applications (DiGA) and their implementation in the German DiGA directory: an analysis

**DOI:** 10.1186/s12913-023-09287-w

**Published:** 2023-04-17

**Authors:** Melanie Mäder, Patrick Timpel, Tonio Schönfelder, Carsta Militzer-Horstmann, Sandy Scheibe, Ria Heinrich, Dennis Häckl

**Affiliations:** 1 Faculty of Economics and Management Science, Leipzig University, Chair for Health Economics and Management, Leipzig, Germany; 2 Scientific Institute for Health Economics and Health System Research (WIG2 GmbH), Markt 8, 04109 Leipzig, Germany; 3 Department of Health Sciences/Public Health, Dresden University, Dresden, Germany

**Keywords:** Digital health applications, DiHA, DiGA, Digital health technology, Evidence evaluation, Evaluation concept

## Abstract

**Background:**

With its digital health application (DiGA)-system, Germany is considered one of Europe's pioneers in the field of evidence-based digital health. Incorporating DiGA into standard medical care must be based on evidence-based success factors; however, a comprehensive overview of the evidence required of scientific studies for their approval is lacking.

**Objective:**

The study aims to, (1) identify specific requirements defined by the Federal Institute for Drugs and Medical Devices (German: Bundesinstitut für Arzneimittel- und Medizinprodukte; BfArM) to design adequate studies, proving a positive healthcare effect, and (2) to assess the evidence given for applications permanently listed in the DiGA directory.

**Methods:**

A multi-step approach was used: (1) identification of the evidence requirements for applications permanently listed in the DiGA directory, (2) identification of the evidence available supporting them.

**Results:**

All DiGA permanently listed in the DiGA directory (13 applications) are included in the formal analysis. Most DiGA addressed mental health (*n* = 7), and can be prescribed for one or two indications (*n* = 10). All permanently listed DiGA have demonstrated their positive healthcare effect through a medical benefit, and most of them provide evidence for one defined primary endpoint. All DiGA manufacturers conducted a randomized controlled trial.

**Discussion:**

It is striking that— although patient-relevant structural and procedural improvements show high potential for improving care, especially in terms of processes — all DiGA have provided a positive care effect via a medical benefit. Although BfArM accepts study designs with a lower level of evidence for the proof of a positive healthcare effect, all manufacturers conducted a study with a high level of evidence.

**Conclusion:**

The results of this analysis indicate that permanently listed DiGA meet higher standards than required by the guideline.

## Background

Digital health applications (known by their German abbreviation, DiGA) are increasingly present, with health systems around the world creating different legal frameworks for their integration into standard care. Together with Belgium, Germany was one of the first countries in Europe to develop an official framework to reimburse DiGA use. Other countries, such as England, Denmark, the Netherlands, Norway, Sweden, and the USA, are still in the development process [1]. France has already announced its intention to adopt the German system [2].

DiGA are certified medical devices with a primarily digital function, through which the medical purpose is achieved. Mainly used by patients, they detect, monitor, treat, or alleviate disease, injury, or disability. Shared use between patients and healthcare providers is also possible, however devices that merely read and transmit data are not considered DiGA [3]. They offer the potential of improved health outcomes [4], higher health standards, and improved and equal access to health services [5]. In addition, DiGA cost-effectively improve patient care [6].

The Federal Republic of Germany’s national parliament passed The Act to Improve Healthcare Provision through Digitalization and Innovation (Digital Healthcare Act – DVG) in 2019. With this law, healthcare providers were given the option of prescribing DiGA. To be part of standard care, the DiGA must be listed in the directory in accordance with paragraph 139e of the fifth book of social code (§ 139e SGB V), first requiring evidence-based proof of a benefit. The benefit is defined by the manufacturer guideline of the Federal Institute for Drugs and Medical Devices (German: Bundesinstitut für Arzneimittel- und Medizinprodukte; BfArM) [3]. First, certification as a medical device in a low-risk class (I or IIa) according to the Medical Device Regulation (MDR) is required. Basic requirements are set for data protection, interoperability, robustness, and user-friendliness, but the central criterion is proof that the DiGA has a positive healthcare effect. DiGA manufacturers must provide this evidence with a comparative scientific study in an International Classification of Diseases (ICD-10)-defined patient group. The positive healthcare effect can be a medical benefit and/or a patient-relevant structural and procedural improvement. The fast-track process offers two paths into the DiGA directory; if all criteria are fulfilled and BfArM reaches a positive decision, the DiGA is permanently included in the DiGA directory; if no positive healthcare effect has yet been demonstrated, the DiGA can be provisional included. In case of the latter, the expected positive healthcare effect must be proven within a period of 12 months with a previously approved evaluation study [3].

The DiGA manufacturer guideline specifies what type of evidence from positive healthcare effect studies is acceptable [3, 7]. After using the fast-track DiGA approval process, Brönneke et al. (2021) highlighted the importance of measures by legislation to help decide if innovations benefit patients in standard healthcare [7]. Similarly, Heimann et al. (2021) described factors required using the fast-track listing, including internal or self-commissioned external audits before submitting the application, consultation with BfArM on positive healthcare effects, and responses to queries from BfArM [8]. Löbker et al. (2021) reported on their experience from consultations on DiGA showing most (80%) of DiGA directory application manufacturers have taken advantage of a consultation during the process. The rate of withdrawn/rejected applications was higher, if manufacturers had not sought advice (63%), compared to manufacturers who had previously discussed key content with BfArM (35%) [9]. Düvel et al. (2021) qualitatively identified potential solutions to improve DiGA access to statutory standard care, recommending a central advisory office [10]. Lantzsch et al. (2022) concluded that there is room for improvement in the fast-track process, particularly in study reporting, as well as in outcomes for patient-relevant improvement of structure and processes. They demand new study designs to pave the way for the use of real-world data [11]. To generate high-quality evidence of DiGA positive healthcare effects, Stern et al. (2022) recommends further research on the impact of missing data, study endpoints, control group, multimodal interventions, study question, equity, generalizability, confounders and fit for purpose [12]. Geier et al. (2021) described different perspectives of the German Digital Health Association on DiGA, arguing the need for accompanying research to address the specific challenges of study design and methods in generating evidence for DiGA [13]. Hemkens et al. (2021) also emphasized the relevance of a robust evidence-based benefit assessment in DiGA approvals, stating that sustainable and efficient DiGA benefit assessment requires continuously adjusted evaluation in everyday care; central to this are randomized study designs that are integrated into standard care [14].

Incorporating DiGA into standard medical care must be based on evidence-based success factors; however, a comprehensive overview of the evidence required of scientific studies for their approval is lacking [15]. We want to investigate which methodologies used to generate evidence-based proof of benefit have been successfully implemented, based on the already permanently listed DiGA.

Our study aims to, (1) identify specific requirements defined by BfArM to design adequate studies, proving a positive healthcare effect, and (2) to assess the evidence given for applications permanently listed in the DiGA directory.

## Methods

All permanently listed applications in the German DiGA directory (as of November 15^th^, 2022) were included in this study.

The study used a multi-step approach: First, we identified the evidence requirements for applications permanently listed in the DiGA directory, based on an analysis of the BfArM manufacturer guideline (as of March 18^th^, 2022) using the PICOS scheme: Population, Intervention, Control, Outcome, Study Design [16]. Then, a single researcher extracted these requirements, and then they were double**-**checked by a second independent researcher. All extracted data were transferred and categorized to a data extraction sheet (MS Excel). Finally, the evidence available supporting the permanently listed DiGA was identified, using the following sources: DiGA directory; study registries (German registry of clinical studies (DRKS), clinical trials.gov, ISRCTN registry); published study protocols; published study reports; submitted publications of permanently listed DiGA; and finally, manufacturer websites.

**Fig. 1:**
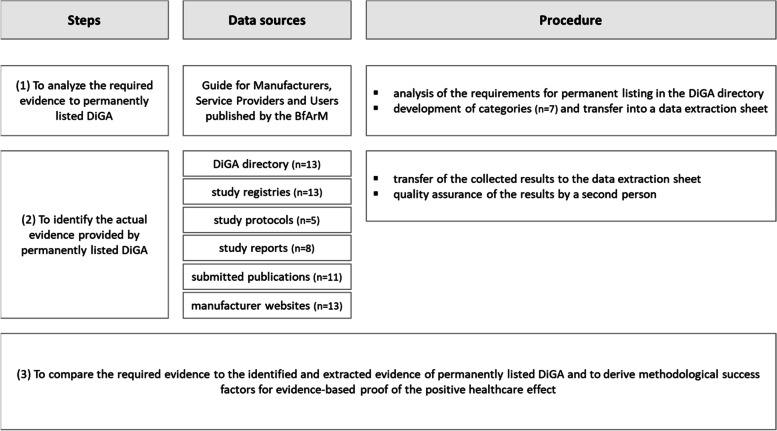
Methodological approach BfArM = Federal Institute for Drugs and Medical Devices (German: Bundesinstitut für Arzneimittel- und Medizinprodukte, DiGA = Digital health application

The sources (including studies, study protocols, reports) were used to extract data using the pre-defined data extraction sheet. Data extraction was performed by one researcher, followed by quality assurance of extracted information carried out by a second researcher. The methodological approach is represented in Fig. 1.

## Results

All DiGA permanently listed in the DiGA directory (13 applications) are included in the formal analysis (Table 1). Most of these were initially accepted into the DiGA directory with an application directly for permanent listing. Four DiGA manufacturers (Kalmeda, Selfapy Depression, Vivira and Zanadio) initially applied for a provisional listing during testing, before being permanently listed. All four applications with initial provisional inclusion in the DiGA directory extended the trial period; Kalmeda by three months, Selfapy Depression and Vivira by four months, and Zanadio by ten months.

**Table 1:** Overview of indication area and quantity of ICD-10-Codes (three, four and five-digit)

DiGA	Indication area	Quantity ICD-10 3 digit	Quantity ICD-10 4 digit	Quantity ICD-10 5 digit
**Deprexis** [17, 18]	mental health	0	6	0
**Elevida** [19]	nervous system	1	0	0
**Hello Better Diabetes and Depression** [20, 21]	hormones and metabolism	2	0	0
**Hello Better Panik** [22, 23]	mental health	0	1	1
**Hello Better Stress and Burnout** [24, 25]	mental health	1	0	0
**Hello Better Vaginismus Plus** [26, 27]	mental health	0	2	0
**Kalmeda** [28]	ears	0	1	0
**Selfapy Depression** [29]	mental health	0	2 *(9)* ^*a*^	0
**Somnio** [30]	mental health	0	1	0
**Velibra** [31]	mental health	0	3	1
**Vivira** [32]	muscles, bones, joints	0	11 *(16)* ^*a*^	9 *(29)* ^*a*^
**Vorvida** [33]	nervous system	0	2	0
**Zanadio** [34]	hormones and metabolism	0	0	2

*DiGA* Digital health application, *ICD* International Classification of Diseases

^a^Originally targeted number of indications

We identified seven relevant categories from our BfArM manufacturer guideline document analysis: (1) patient population, (2) positive healthcare effect and study endpoints, (3) study design, (4) study location, (5) observation period and observation times, (6) sample size and drop-out, and (7) study results. These categories are used to guide the step-by-step reporting of findings.

### Patient population and patient characteristics

A positive healthcare effect must be provided for at least one defined patient population. The delineation is based on one or more indications listed in the ICD-10 catalog; this international classification provides a specific disease definition. Three- or four-digit ICD-10 codes are permissible. This can be used to distinguish whether the positive healthcare effect should be demonstrated for all patients with a specific disorder (e.g., F32.-Depressive Episode) or for a specific patient population (e.g., F32.0-Mild Depressive Episode). If several indications are given, the evidence must be provided for each defined patient group. Thus, the ICD-10 code chosen is a measure of the specificity and concreteness of the patient population addressed. If the positive healthcare effect among the indications is comparable, the proof can be pro-vided for several indications combined [3].

Of 13 DiGA applications, seven address indications in mental health. One DiGA works at the interface of mental health and metabolism (Hello Better Diabetes and Depression), and the others are relevant to the nervous system (Elevida, Vorvida), the musculoskeletal system (Vivira), ears (Kalmeda), and hormones and metabolism (Zanadio). The majority (*n* = 10) of the listed DiGA can be prescribed for one or two indications, except for Deprexis with six, Velibra with four and Vivira with 20 indications. The manufacturers of Selfapy Depression originally sought listing for nine indications but were only able to prove a positive healthcare effect for two of these. Vivira manufacturers were able to provide evidence of a positive healthcare effect for 20 of the 45 indications they originally sought listing for. Table 1 shows the DiGA indication areas addressed, and the number of ICD-10 codes (separated into three-, four-, and five-digit codes) for which listing in the DiGA directory was achieved.

Except for Hello Better Vaginismus Plus, all permanently listed DiGA were developed for both women and men. In all studies, the proportion of female participants was higher than that of men. This trend was evident in both intervention groups (IG) and control groups (CG). Zanadio was approved for women only, due to an insufficient number of males in the trial. All DiGA manufacturers defined a minimum age of 18 years as inclusion criterion. On average, the study participants’ mean age was between 28.0 (Hello Better Vaginismus Plus) and 51.3 (Hello Better Diabetes and Depression) years. In all studies already published (except for Hello Better Vaginismus Plus) the average age of the participants was between 35 and 55 years. There were no significant differences in age between IG and CG. To date, participants with a higher education and a permanent job are more frequently represented in DiGA studies (Table 2).

**Table 2:** Overview of patient population and patient characteristics

DiGA	Patient population		Patient characteristics					
	**Gender**	**Age**	**Gender**		**Age**		**Education and/or employment status**	
			**IG**	**CG**	**IG**	**CG**	**IG**	**CG**
**Deprexis**								
Study 1 [17]	m / f	18-65 years	f=58 (74.4 %) m=20 (25.6 %)	f=64 (75.3 %) m=21 (24.7 %)	M=44 (11.02)	M=40 (11.48)	Educational status: Lower secondary=4 (5.1 %) Middle secondary=23 (29.5 %) Higher secondary=14 (17.9 %) Highest secondary=37 (47.4 %) Employment status: Full time=38 (48.7 %) Regular part-time=9 (11.5 %) Not working=31 (39.7 %)	Educational status: Lower secondary=3 (3.5 %) Middle secondary=14 (16.5 %) Higher secondary=12 (14.1 %) Highest secondary=53 (62.4 %) Employment status: Full time=31 (36.5 %) Regular part-time=16 (18.8 %) Not working=38 (44.7 %)
Study 2 [18]	m / f	18-65 years	f=350 (68.8 %) m=159 (31.2 %)	f=345 (68.5 %) m=159 (31.5 %)	M=42.8 (11.0)	M=42.9 (11.0)	Educational status: Not yet completed=2 (0.4 %) No degree=1 (0.2 %) Lower secondary school=29 (5.7 %) Middle secondary school=131 (25.8 %) Higher secondary school qualifying for university of applied science=87 (17.1 %) Higher secondary school qualifying for university=249 (48.9 %) Other=10 (2.0 %) Employment status: Full time=220 (43.3 %) Part time=117 (23.0 %)	Educational status: Not yet completed=0 (0.0 %) No degree=0 (0.0 %) Lower secondary school=24 (4.8 %) Middle secondary school=112 (22.2%) Higher secondary school qualifying for university of applied science=85 (16.8 %) Higher secondary school qualifying for university=271 (53.8%) Other=12 (2.4 %) Employment status: Full time=214 (42.4 %) Part time=114 (22.6 %)
**Elevida** [19]	m / f	≥18 years	f=114 (82.0 %) m=25 (18 %)	f=108 (79 %) m=28 (21 %)	M=40.8 (11.1)	M=41.9 (9.4)	Employment status: Full-time/part-time=71 (51 %) Housemaker/student/other=13 (9 %) Unemployed/unable to work/retired= 55 (40 %)	Employment status: Full-time/part-time=64 (47 %) Housemaker/student/other=11 (8 %) Unemployed/unable to work/retired=61 (45 %)
**Hello Better Diabetes and Depression** [20, 21]	m / f	≥18 years	f=81 (63.3%) m=47 (36.7 %)	f=79 (62.7%) m=47 (37.3 %)	M=50.2 (11.6)	M=51.3 (11.9)	Educational status: Low=19 (14.8 %) Middle=76 (59.4 %) High=32 (25.0 %) Employment status: Employed=77 (60.2 %)	Educational status: Low=17 (13.5 %) Middle=64 (50.8 %) High=45 (35.7 %) Employment status: Employed=79 (62.7 %)
**Hello Better Panik** [22, 23]	m / f	>18 years	f=27 (60 %) m=18 (40 %)	f=24 (51.1 %) m=23 (48.9 %)	M=39.33 (10.83)	M=37.43 (10.03)	Educational status: Low=3 (6.7 %) Middle=11 (24.4 %) High=31 (68.9 %) Employment status: Full-time=27 (60.0 %) Part-time=15 (33.3 %) Not employed=3 (6.7 %) Unemployed=0 Currently sick=0	Educational status: Low=4 (8.5 %) Middle=14 (29.8 %) High=29 (61.7 %) Employment status: Full-time=25 (53.2 %) Part-time=15 (31.9 %) Not employed=3 (6.4 %) Unemployed=1 (2.1 %) Currently sick=3 (6.4 %)
**Hello Better Stress and Burnout** [24, 25]	m / f	≥18 years	f=97 (73.5 %) m=35 (26.5 %)	f=96 (72.7 %) m=36 (27.3 %)	M=42.4 (10.7)	M=44.2 (9.6)	Educational status: Low=3 (2.3 %) Middle=25 (18.9 %) High=104 (78.8 %) Employment status: Full-time=105 (79.5 %) Part-time=25 (18.9 %) Currently sick=2 (1.5 %)	Educational status: Low=2 (1.5 %) Middle=31 (23.5 %) High=99 (75.0 %) Employment status: Full-time=99 (75.0 %) Part-time=32 (24.2 %) Currently sick=1 (0.8 %)
**Hello Better Vaginismus Plus** [26, 27]	f	≥18 years	f=100 (100 %) m=0	f=100 (100 %) m=0	M=29.46 (9.82)	M=28.04 (7.84)	Educational status: Low=0 (0.00 %) Middle=42 (42.00 %) High=58 (58.00 %)	Educational status: Low=4 (4.00 %) Middle=47 (47.00 %) High=49 (49.00 %)
**Kalmeda** [28]	m / f	≥18 years	f= 49 (52.1%) m=45 (47.9%)	f=41 (44.1%) m=52 (55.9%)	M=48.1 (12.8)	M= 48.4 (12.2)	n.r.	n.r.
**Selfapy Depression** [29]	m / f	18-65 years	IG1: f=126 (83.4 %) m=25 (16.6 %) IG2: f=126 (84.0 %) m=24 (16.0 %)	f=81 (81.0 %) m=19 (19.0 %)	IG1: M=38 (10.7) IG2: M=37 (10.8)	M=36 (11.9)	Employment status: Employee: IG 1=82 (54.3 %), IG2=86 (57.3 %) Self-employed: IG1=3 (2.0 %), IG2=4 (2.7 %) Trainee: IG1=12 (7.9 %), IG2=6 (4.0 %) Other: IG1=7 (4.6 %), IG2=3 (2.0 %) Not reported: IG1=47 (31.3 %), IG2=51 (34.0 %)	Employment status: Employee=57 (57.0 %) Self-employed=2 (2.0 %) Trainee=25 (25.0 %) Other=14 (14.0 %) Not reported=2 (2.0 %)
**Somnio** [30]	m / f	≥18 years	f=21 (72 %) m=8 (28 %)	f=18 (67 %) m=9 (33 %)	M=41.72 (17.31)	M=44.04 (20.05)	Educational status: Obligatory school=1 (3 %) Basic apprenticeshi*p=*3 (10 %) Higher apprenticeshi*p=*6 (21 %) College=5 (17 %) University=11 (38 %) Other=3 (10 %) Employment status: Self-employed=3 (10 %) Employee=17 (59 %) Retired=4 (14 %) Housewife/ma*n=*1 (3 %) Unemployed=2 (7 %) Other=2 (7 %)	Educational status: Obligatory school=2 (7 %) Basic apprenticeshi*p=*5 (19 %) Higher apprenticeshi*p=*1 (4 %) College=4 (15 %) University=11 (41 %) Other=4 (15 %) Employment status: Self-employed=3 (11 %) Employee=13 (48 %) Retired=4 (15 %) Housewife/ma*n=*2 (7 %) Unemployed=3 (11 %) Other=2 (7 %)
**Velibra** [31]	m / f	≥18 years	f=48 (69 %) m=22 (31 %)	f=50 (72 %) m=19 (28 %)	M=42.1 (12.2)	M=41.8 (12.2)	Educational status: Compulsory school=5 (7 %) Apprenticeshi*p=*32 (46 %) College=9 (13 %) University=24 (34 %) Employment status: Full-time paid work=22 (31 %) Part-time paid work=21 (30 %) Unemployed =13(19 %) At-home parent=2 (3 %) Student=2 (3 %) Retired=10 (14 %)	Educational status: Compulsory school=4 (6 %) Apprenticeshi*p=*25 (36 %) College=10 (14 %) University=30 (43 %) Employment status: Full-time paid work=27 (39 %) Part-time paid work=13 (19 %) Unemployed=11 (16 %) At-home parent=3 (4 %) Student=5 (7 %) Retired=10 (15 %)
**Vivira** [32]	m / f	≥18 years	*No data published yet*					
**Vorvida** [33]	m / f	≥18 years	f=170 (56 %) m=136 (44 %)	f=149 (49 %) m=153 (51 %)	M=40.4 (11.2)	M=40.7 (12.1)	Educational status: Apprenticeship/technical school=203 (66.3 %) University/college=78 (25.5 %) No vocational training=17 (5.6 %) Other=8 (2.6 %) Employment status: Employed=236 (77.1 %) Not employed=45 (14.7 %) Learning=15 (4.9 %) Other=10 (3.3 %)	Educational status: Apprenticeship/technical school=185 (61.3 %) University/college=97 (32.1 %) No vocational training=18 (6.0 % Other=2 (0.7 %) Employment status: Employed=239 (79.1 %) Not employed=41 (13.6 %) Learning=20 (6.6 %) Other=2 (0.7 %)
**Zanadio** [34]	m / f	18-65 years	f=n.r m=7	f=n.r. m=6	*No data published yet*			

*DiGA* Digital health application, *IG* Intervention group, *CG* Control group, *m* Male, *f* Female, *M (SD)* Mean (standard deviation)

### Positive healthcare effect and study endpoints

For permanent or provisional inclusion in the DiGA directory, manufacturers must demonstrate one or more positive healthcare effects by means of a scientific study. This proof can be provided as a medical benefit (improvement of the state of health, reduction of the duration of the disease, prolongation of survival, or improvement in the quality of life), and/or a patient-relevant improvement of structure and processes (e.g., coordination of treatment processes, alignment of treatment with guidelines and recognized standards, or adherence). The positive healthcare effect to be demonstrated must relate directly to the insured person and be proven within the framework of the study to be conducted by means of defined endpoints [3].

All permanently listed DiGA have demonstrated their positive healthcare effect through a medical benefit. The manufacturers of Velibra and Vorvida demonstrated a patient-relevant structural and procedural improvement, in addition to the medical benefit. The most frequently proven medical benefit of all permanently listed DiGA can be categorized as improvement in the state of health. The Vivira application manufacturers additionally aimed for a reduced disease duration and an improved quality of life, but were unable to provide evidence for either. The Velibra manufacturers demonstrated a patient-relevant improvement of structure and process, reducing therapy-related efforts of patients and their relatives, and the Vorvida manufacturers, an improvement in patient autonomy (Table 3). Most DiGA provide evidence for one defined primary endpoint, except the Velibra manufacturers, who defined a total of five primary endpoints and proved four of them (the subscale, mental health, of the Short Form Health Survey-12, could not be proven). Depending on the defined patient group, various primary endpoints are attempted. The operationalization of the primary endpoints was carried out using validated questionnaires (Table 5).

**Table 3:** Overview of positive healthcare effects, indications, study designs, observation durations, sample sizes, IG, CG

DiGA	Positive care effect	Medical benefit	Patient-relevant improvement of structure and processes	Study design	Observation duration and survey times	Sample size	IG	CG	Location of study	Time period of study^b^
**Deprexis**	medical benefit	improvement in the state of health	n.a	RCT	**3 months** t1 = 3 months t2 = 6 months follow-up		DiGA + UC UC + waiting group			
Study 1 [17]						***n*** **= 163** IG = 78 CG = 85			Hamburg, Lübeck, Bern	02/2013–05/2014
Study 2 [18]						***n*** **= 1013** IG = 509 CG = 504			Berlin, Bielefeld, Hamburg, Lübeck	07/2012–02/2017
**Elevida** [19]	medical benefit	improvement in the state of health	n.a	RCT	**12 weeks** t1 = 12 weeks t2 = 24 weeks follow-up	***n*** **= 275** IG = 139 CG = 136	DiGA + UC	UC + waiting group	Hamburg	07/2014–05/2016
**Hello Better** **Diabetes and Depression** [20, 21]	medical benefit	improvement in the state of health	n.a	RCT	**8 weeks** t1 = 8 weeks t2 = 6 months follow-up 1 t3 = 12 months follow-up 2	***n*** **= 254** IG = 128 CG = 126	DiGA + UC	UC + waiting group + online program for knowledge transfer	Lüneburg	03/2013–01/2015
**Hello Better** **Panik** [22, 23]	medical benefit	improvement in the state of health	n.a	RCT	**8 weeks** t1 = 8 weeks t2 = 3 months follow-up 1 t3 = 6 months follow-up 2	***n*** **= 92** IG = 45 CG = 47	DiGA + UC	UC + waiting group	Lüneburg	08/2013–05/2015
**Hello Better** **Stress and Burnout** [24, 25]	medical benefit	improvement in the state of health	n.a	RCT	**7 weeks** t1 = 7 weeks t2 = 6 months follow-up 1 t3 = 12 months follow-up 2, only IG	***n*** **= 264** IG = 132 CG = 132	DiGA + UC	UC + waiting group	Lüneburg	03/2013–11/2014
**Hello Better** **Vaginismus Plus** [26, 27]	medical benefit	improvement in the state of health	n.a	RCT	**12 weeks** t1 = 12 weeks t2 = 6 months follow-up	***n*** **= 200** IG = 100 CG = 100	DiGA + UC	UC + waiting group	Erlangen	04/2016–9/2018
**Kalmeda** [28]	medical benefit	improvement in the state of health	n.a	RCT	**9 months (IG)** **12 months (CG)** t1 = 3 months t2 = 9 months (IG) t3 = 12 months (CG)	***n*** **= 187** IG = 94 CG = 93	DiGA	waiting group + general information about tinnitus	Duisburg	08/2020–04/2022
**Selfapy** **Depression** [29]	medical benefit	improvement in the state of health	n.a	RCT	**12 weeks** t1 = 6 weeks t2 = 12 weeks t3 = 24 weeks follow-up	***n*** **= 401** IG1 = 151 IG2 = 150 CG = 100	IG1: DiGA with psychological support (proof not provided) IG2: DiGA without any support	waiting group + weekly standardized mindfulness exercises via email	Berlin	08/2019-n.r
**Somnio** [30]	medical benefit	improvement in the state of health	n.a	RCT	**6 weeks** t1 = 6 weeks t2 = 12 months follow-up	***n*** **= 56** IG = 29 CG = 27	DiGA	waiting group	Zurich	01/2016–10/2016
**Velibra** [31]	medical benefit + patient-relevant improvement of structure and processes	improvement in the state of health	reduction of therapy-related efforts and strains for patients and their relatives	RCT	**9 weeks** t1 = 9 weeks t2 = 6 months follow-up	***n*** **= 139** IG = 70 CG = 69	DiGA + UC	UC + waiting group	Bern	05/2014–05/2016
**Vivira** [32]	medical benefit	improvement in the state of health	n.a	RCT	**12 weeks** t1 = 2 weeks t2 = 6 weeks t3 = 12 weeks	***n*** ** = 215**	DiGA	UC (physiotherapy)	Tübingen	11/2020–03/2021
				pre-post study^a^	**12 weeks**	hi*p* = 256 knee = 402				
**Vorvida** [33]	medical benefit + patient-relevant improvement of structure and processes	improvement in the state of health	patient autonomy	RCT	**6 months** t1 = 3 months t2 = 6 months	***n*** **= 608** IG = 306 CG = 302	DiGA + UC	UC + waiting group	Hamburg	03/2015-ongoing
**Zanadio** [34]	medical benefit	improvement in the state of health	n.a	RCT	**9 months** t1 = 3 months t2 = 6 months t3 = 9 months t4 = 12 months follow-up	***n*** ** = 149**	DiGA	UC	online in Germany	02/2021-ongoing

*DiGA* Digital health application, *IG* Intervention group, *CG* Control group, *RCT* Randomized controlled trial, *UC* Usual care, *n.a*. Not applicable

### Study design

A quantitative comparative (retrospective or prospective) study is required to provide evidence of a positive healthcare effect. Studies intended to prove a positive healthcare effect should reflect the actual healthcare reality and be conducted with data close to the healthcare system. A comparison can be made intra-individually or inter-individually. In an intra-individual, single-arm comparison, the data can, for example, be collected before and after the application of the DiGA. In an inter-individual, two-arm comparison, the intervention group data (DiGA use) are compared with control group data. The following options exist for the design of the control group: (1) treatment without the use of a DiGA, (2) non-treatment, and (3) treatment with another, comparable DiGA already permanently listed in the DiGA directory at the time of application. It is important that the selection of the control group corresponds to the standard of care [3].

All permanently listed DiGA manufacturers conducted a randomized controlled trial (RCT) using an inter-individual comparison of an intervention group with a control group. Almost all DiGA were tested against standard of care. The manufacturers of Selfapy Depression originally designed two intervention groups: one with, and one without psychological support. Evidence could only be proved for DiGA use without psychological support. Most control groups (standard of care) were offered access to the DiGA after the end of the study. Three DiGA manufacturers provided additional materials to the participants: Hello Better Diabetes and Depression enabled the control group to use an online knowledge transfer program, Kalmeda provided general information on the topic of tinnitus, and the manufacturers of Selfapy Depression sent weekly emails with standardized mindfulness exercises (Table 3).

### Study location

Studies to prove a positive healthcare effect must be conducted in Germany, as the actual healthcare setting is closely linked to the positive healthcare effects. The comparison of the intervention against a control group without DiGA use is only meaningful, if a treatment in the German healthcare system is addressed. If the comparability of the healthcare situation can be proven, a study in other countries is also permissible [3].

Evidence of positive healthcare effect was obtained for 11 DiGA in Germany, and for two applications in Switzerland (Table 3).

### Observation period and observation times

There are no concrete specifications of observation periods and times in the BfArM guideline. Baseline data collection is considered reasonable; the data collection periods (incl. possible follow-ups after the intervention phase) should be described [3].

The observation periods ranged from six weeks (Somnio) to nine months (Kalmeda, Zanadio). Most DiGA studies had an observation period of eight to twelve weeks. In addition to collecting data at baseline (t0) and at the fixed primary endpoint collection time, ten DiGA studies also collected follow-up data. In most cases, follow-up data were collected after six and/or twelve months. Almost all studies date back several years, so that existing data was used to prove a positive healthcare effect (Table 3).

### Sample size and drop-out

BfArM requires studies to have an adequate sample size calculation/planning. In confirmatory studies, the sample sizes should be estimated based on the primary outcome measure and the relevant effect size. BfArM also requires studies to record the number of drop-outs and the respective reasons for these, with a transparent presentation in a study flow chart. A high drop-out rate should also be considered in connection with the respective clinical picture. Studies on addictive diseases, for example, show higher drop-out rates in actual care reality compared to diseases with recognized therapies and high responder rates. A high drop-out rate does not indicate the success of the application and can only be accepted to a limited extent [3].

The manufacturers of Somnio were able to provide evidence of a positive healthcare effect with the lowest sample size (*n* = 56), and the manufacturers of Deprexis used the largest sample (*n* = 1,013). Proof was provided for Hello Better Panik based on 92 cases. Five studies had sample sizes between 100 and 199, and six studies between 200 and 299. All other studies worked with sample sizes larger than 300. The distribution of the individuals to the intervention and control groups was done by means of 1:1 randomization in most studies; one exception was the Selfapy Depression study, with two intervention groups (IG1 = 151, IG2 = 150, CG = 100). The drop-out rates at the time of the primary endpoint survey ranged from 6.7% (Zanadio) to 30.0% (Vorvida). In the pre-post study of Vivira (intended to transfer the results to other indications), drop-out rates of more than 90% were observed, which is why the evidence was assessed as not provided. Drop-out rates were higher in intervention groups than in control groups, except for two studies (for Hello Better Panik and Selfapy Depression applications). Drop-out rates at the last follow-up time point ranged from 5.9% (Somnio, a respondent who missed t1 provided data at t2) to 61.3% (Selfapy Depression) (Table 4).

**Table 4:** Overview of sample sizes and drop-outs

DiGA	Case number			Drop-out at primary survey time point			Total drop-out at the end of the study		
	**total**	**IG**	**CG**	**total**	**IG**	**CG**	**total**	**IG**	**CG**
**Deprexis**									
Study 1 [17]	163	78	85	29 (17.8%)	17	12	45 (27.6%)	21	24
Study 2 [18]	1013	509	504	219 (21.6%)	114	105	259 (25.6%)	131	128
**Elevida** [19]	275	139	136	51 (18.5%)	36	15	64 (23.3%)	44	20
**Hello Better** **Diabetes and Depression** [20, 21]	254	128	126	47 (18.5%)	31	16	74 (29.1%)	50	24
**Hello Better** **Panik** [22, 23]	92	45	47	8 (8.7%)	4	4	20 (21.7%)	10	10
**Hello Better** **Stress und Burnout** [24, 25]	264	132	132	21 (8.0%)	16	5	51 (19.3%)	40	11
**Hello Better** **Vaginismus Plus** [26, 27]	200	100	100	30 (15.0%)	22	8	51 (25.5%)	42	9
**Kalmeda** [28]	187	94	93	24 (12.8%)	16	8	92 (48.9%)	41	51
**Selfapy Depression** [29]	401	IG1 = 151 IG2 = 150	100	95 (23.7%)	IG1 = 19 IG2 = 30	46	246 (61.3%)	IG1 = 87 IG2 = 88	71
**Somnio** [30]	56	29	27	4 (7.1%)	4	0	3 (5.4%)	3	0
**Velibra** [31]	139	70	69	19 (13.7%)	13	6	26 (37.0%)	26	n.a
**Vivira** [32]									
RCT	215	2	n.r	n.r	n.r	n.r	n.r	n.r	n.r
Pre-Post-Study	hi*p* = 256 knee = 402	n.r	n.r	hi*p* = 242 (94.5%) knee = 372 (92.5%)	n.r	n.r	n.r	n.r	n.r
**Vorvida** [33]	608	306	302	183 (30.0%)	114	69	183 (30.1%)	114	69
**Zanadio** [34]	149	n.r	n.r	10 (6.7%)	n.r	n.r	n.r	n.r	n.r

DiGA = Digital health application, IG = Intervention group, CG = Control group, n.r. = Not reported, n.a. = Not applicable

### Study results

A positive healthcare effect is considered proven if the outcome is (clinically) relevant, patient-relevant, and statistically significant [3].

All studies were able to deliver significant differences between the intervention and control groups at the primary endpoint and thus, significant results. The effect sizes, according to Cohen et al. were small effect sizes (< 0.5) for three DiGA; medium effect sizes (0.5–0.8) for six applications, and in the range of large effect sizes (> 0.8) for four DiGA. No effect sizes were reported for four DiGA. For the Deprexis study, the effect sizes of the two published studies were used, and for the Velibra study, the effect sizes were those related to the four primary endpoints. The effect sizes increased for five DiGA at the follow-up time point, and decreased for two DiGA. No effect sizes at the follow-up time point were reported for six DiGA. DiGA that reported the minimum clinically relevant difference also exceeded this effect size (Table 5).

**Table 5:** Overview of primary endpoints and study results (based on the intention-to-treat analyses)

DiGA	Positive care effect	Primary endpoint	Operationalization	Interpretation	MCID	Results			
**Deprexis**						t0 (Baseline)	t1 (3 months)^a^	t2 (6 months follow-up)	
Study 1 [17]	improvement of the state of health	depression severity	Patient Health Questionnaire-9 (PHQ-9)	0–27 higher scores indicate stronger symptoms level of depression: 0–4 = minimal 5–9 = mild 10–14 = moderate 15–19 = moderately severe 20–27 = severe [35]	5-point reduction [35]	IG: M = 16.62 (3.44) CG: M = 17.20 (3.86)	IG: M = 10.08 (6.37) CG: M = 13.64 (6.14) ***p*** **< 0.01** Cohens d = 0.57 [0.22, 0.92]	IG: M = 11.28 (6.04) CG: M = 13.39 (6.59) Cohens d = 0.33 [0.3, 0.69]	
Study 2 [18]						IG: M = 10.23 (2.41) CG: M = 10.34 (2.40)	IG: M = 7.54 (4.04) CG: M = 9.15 (4.30) Cohens d = 0.39 [0.13, 0.64]	IG: M = 7.31 (4.18) CG: M = 8.69 (4.41) Cohens d = 0.32 [0.06, 0.59]	
**Elevida** [19, 36]						**t0 (Baseline)**	**t1 (12 weeks)** ^a^	**t2 (24 weeks follow-up)**	
	improvement of the state of health	fatigue	Chalder Fatigue Scale	0–33 higher scores indicate stronger symptoms [37]	between 0.7 and 1.4 [37, 38]	IG: M = 21.58 (5.32) CG: M = 21.17 (5.02)	Intergroup difference -2.74 [-1.16, -4.32] ***p*** **= 0.0007** Cohens d = 0.53	Intergroup difference -2.19 [-0.57, -3.82] ***p*** **= 0.0080**	
**Hello Better** **Diabetes and Depression** [20, 21]						**t0 (Baseline)**	**t1 (8 weeks)** ^a^	**t2 (6 months follow-up 1)**	**t3 (12 months follow-up 2)**
	improvement of the state of health	depressive symptom severity	Allgemeine Depressionsskala (ADS)	0–60 higher scores indicate stronger symptoms [39] score of 23 or higher is indicative of clinically relevant depressive symptoms in German populations [40]	standardized mean difference ≥ 0.24 [41]	IG: M = 32.22 (6.96) CG: M = 31.55 (7.57)	IG: M = 20.86 (9.73) CG: M = 29.1 (9.08) MD = 8.07 (1.37) ***p*** **< 0.001** Cohens d = 0.94 [0.62, 1.25]	IG: M = 20.09 (11.15) CG: M = 27.13 (10.14) MD = 5.78 (1.95) ***p*** **< 0.001** Cohens d = 0.62 [0.21, 1.04]	IG: M = 19.88 (10.15) KG: M = 26.73 (10.43) MD = 5.57 (2.02) ***p*** **= 0.002** Cohens d = 0.63 [0.18, 1.08]
**Hello Better** **Panik** [22, 23]						**t0 (Baseline)**	**t1 (8 weeks)** ^a^	**t2 (6 months follow-up 1)**	**t3 (12 months follow-up 2)**
	improvement of the state of health	severity of panic and agoraphobia symptoms	Panic Symptom Severity and Self-Rating (German: PAS)	0–52 higher scores indicate stronger symptoms 0–8 = no clinically relevant symptoms 9–28 = moderate symptoms > 29 = severe level of symptoms [42]		IG: M = 18.18 (6.54) CG: M = 19.43 (5.49)	IG: M = 11.73 (6.90) CG: M = 17.60 (8.86) MD = 5.99 (1.86) ***p*** **= 0.009** Cohens d = 0.78 [0.31, 1.26]	IG: M = 9.66 (7.65) CG: M = 16.32 (7.13) MD = 6.72 (1.97) ***p*** **= 0.009** Cohens d = 1.00 [0.42, 1.58]	IG: M = 8.63 (6.33) CG: M = 14.68 (7.65) MD = 6.07 (1.90) ***p*** **= 0.009** Cohens d = 0.96 [0.37, 1.56]
**Hello Better** **Stress and Burnout** [24, 25]						**t0 (Baseline)**	**t1 (7 weeks)** ^a^	**t2 (6 months follow-up 1)**	**t3 (12 months follow-up 2)**
	improvement of the state of health	level of perceived stress	Perceived Stress Scale (PSS-10)	0–40 higher scores indicate stronger symptoms 0–13 = low 14–26 = moderate 27–40 = high perceived stress [43]		IG: M = 25.89 (3.85) CG: M = 25.15 (3.96)	IG: M = 17.88 (6.17) CG: M = 22.96 (6.07) ***p*** **< 0.001** Cohens d = 0.83 [0.58–1.08]	IG: M = 16.08 (6.03) CG: M = 22.10 (5.81) ***p*** **< 0.001** Cohens d = 1.02 [0.76–1.27]	IG: M = 16.25 (6.35)
**Hello Better** **Vaginismus Plus** [26, 27]						**t0 (Baseline)**	**t1 (12 weeks)** ^a^	**t2 (6 months follow-up)**	
	improvement of the state of health	intercourse penetration behavior	Primary Endpoint Questionnaire (PEQ)	0 = not attempted or attempted, but unsuccessful 1 = attempted and sometimes successful or attempted and always successful [44]		IG: M = 0 CG: M = 0	IG = 31 (31%) CG = 13 (13%) Chi-Quadrat = 9.44 ***p*** **< 0.01** OR = 3.01 [1.46–6.18]	IG = 29 (29%) CG = 20 (20%) Chi-Quadrat = 2.19 ***p*** **= 0.19** OR = 1.63 [0.85–3.14]	
**Kalmeda **[28]						**t0 (baseline)**	**t1 (3 months)** ^a^	**t2 (9 months) only IG**	**t3 (12 months) only CG**
	improvement of the state of health	tinnitus exposition	Tinnitus questionnaire according to Göbel and Hiller	0–84 higher scores indicate stronger symptoms 0–30 = mild 31–46 = medium 47–59 = severe 60–84 = severely [45]		IG: M = 37.14 (13.01) CG: M = 37.46 (14.29)	IG: M = 23.43 (11.77) CG: M = 24.76 (15.71) ***p*** **< 0.0001**	IG: M = 17.61 (10.21) ***p*** **< 0.0001**	CG: M = 20.27 (13.01) ***p*** **< 0.0001**
**Selfapy** **Depression** [29]						**t0 (Baseline)**	**t1 (6 weeks)**	**t2 (12 weeks)** ^a^	**t3 (24 weeks follow-up)**
	improvement of the state of health	depressive symptoms	Beck-Depressions-Inventar II (BDI-II)	0–63 higher scores indicate stronger symptoms 0–8 = no depression 9–13 = minimal depression 14–19 = mild depression 20–28 = moderate depression 29–63 = severe depression [46, 47]		IG1: M = 30.09 (9.18) IG2: M = 30.54 (8.53) CG: M = 30.88 (10.74)	IG1: M = 20.71 (6.98) IG2: M = 22.51 (7.83) CG: M = 29.09 (6.39) IG1 vs. IG2: Cohens d = 0.24 [0.02, 0.48] IG1 vs. CG: Cohens d = 1.25 [0.99, 1.54] IG2 vs. CG: Cohens d = 0.92 [0.65, 1.20]	IG1: M = 16.61 (9.55) IG2: M = 18.49 (8.88) CG: M = 30.26 (6.97) IG1 vs. IG2: Cohens d = 0.20 [− 0.04, 0.45] ***p*** **= 0.18** IG1 vs. CG: Cohens d = 1.63 [1.37, 1.93] ***p*** **< 0.001** IG2 vs. CG: Cohens d = 1.47 [1.22, 1.73] ***p*** **< 0.001**	*for the 24-week follow-up assessment only per protocol data were used*
**Somnio** [30]						**t0 (Baseline)**	**t1 (6 weeks)** ^a^	**t2 (12 months follow-up)**	
	improvement of the state of health	insomnia	Insomnia severity index (ISI)	0–28 higher scores indicate stronger symptoms 0–7 = normal 8–14 = sub-threshold insomnia 15–21 = moderate insomnia > 21 = severe clinical insomnia [48]		IG: M = 15.38 (3.74) CG: M = 13.26 (3.29)	IG: M = 7.80 (4.94) CG: M = 12.04 (3.86) t = 5.06 ***p*** **< 0.001** Cohens d = 1.79	IG: M = 7.36 (5.38) Cohens d = 1.98 [1.31, 2.66]	
**Velibra** [31, 49]						**t0 (Baseline)**	**t1 (9 weeks)** ^a^	**t2 (6 months follow-up)**	
	improvement of the state of health	depression, anxiety and tension/stress	Depression Anxiety Stress Scales – Short Form (DASS-21)	0–120 higher scores indicate stronger symptoms [50]	Δ = 6.42 [51] d = 0.24 [41]	IG: M = 58.2 (24.4) CG: M = 55.8 (21.3)	IG: M = 40.9 (25.7) CG: M = 52.7 (24.7) ***p*** **< 0.01** Cohens d = 0.47 [0.13–0.81]	IG: M = 41.9 (30.0)	
	reduction of therapy-related efforts and strains for patients/their relatives	general psycho- pathology	Brief Symptom Inventory (BSI)	Short version of the Symptom Checklist-90 (SCL-90)		IG: M = 1.34 (0.56) CG: M = 1.27 (0.57)	IG: M = 0.94 (0.63) CG: M = 1.18 (0.71) ***p*** **< 0.001** Cohens d = 0.42 [0.08–0.75]	IG: M = 0.97 (0.77)	
		anxiety-related symptoms	Beck Anxiety Inventory (BAI)	0–63 higher scores indicate stronger symptoms 0–21 = low anxiety 22–35 = moderate anxiety > 36 = potentially concerning levels of anxiety [46]		IG: M = 34.9 (9.1) CG: M = 33.3 (10.3)	IG: M = 27.8 (9.1) CG: M = 31.4 (10.0) ***p*** **< 0.001** Cohens d = 0.41 [0.07–0.74]	IG: 26.6 (9.4)	
		depressive symptoms	Beck Depression Inventory-II (BDI-II)	0–63 higher scores indicate stronger symptoms 0–8 = no depression 9–13 = minimal depression 14–19 = mild depression 20–28 = moderate depression 29–63 = severe depression [46, 47]		IG: M = 22.6 (10.6) CG: M = 22.0 (11.0)	IG: M = 15.8 (12.4) CG: M = 22.9 (12.6) ***p*** **< 0.001** Cohens d = 0.61 [0.27–0.95]	IG: M = 16.3 (13.7)	
		quality of life	SF-12 Mental Health	0–100 higher scores indicate higher quality of life [52]		IG: M = 31.2 (8.8) CG: M = 33.2 (9.5)	IG: M = 37.5 (11.8) CG: M = 33.0 (9.2) ***p*** **< 0.001** Cohens d = 0.49 [0.15–0.83]	IG: M = 39.9 (12.2)	
			SF-12 Physical Health	0–100 higher scores indicate higher quality of life [52]		IG: M = 48.5 (11.2) CG: M = 48.3 (10.8)	IG: M = 48.3 (11.4) CG: M = 47.2 (9.5) ***p*** **= 0.19** Cohens d = 0.16 [− 0.17–0.50]	IG: M = 48.6 (11.1)	
**Vivira** [32]						**t0 (Baseline)**	**t1 (2 weeks)**	**t2 (6 weeks)**	**t3 (12 weeks)** ^a^
	improvement of the state of health	back, knee and hip pain	visual numeric rating scale					difference in change from baseline = -2.34 [-2.84; -1.83] ***p*** **< 0.0001**	difference in change from baseline = -2.44 [-2.92; -1.95] ***p*** **< 0.0001**
**Vorvida** [33]						**t0 (Baseline)**	**t1 (12 weeks)** ^a^	**t2 (6 months)**	
	improvement of the state of health	daily average consumption of pure alcohol in grams)	Quantity-frequency index: self-reported quantities of alcohol in g (last 30 days)			IG: M = 63.69 (61.84) CG: M = 61.64 (58.84)	IG: mM = 40.8 (3.3) CG: mM = 56.8 (3.3) ***p*** **= 0.001** Cohens d = 0.278	IG: mM = 32.3 (2.1) CG: mM = 44.1 (2.1) ***p*** **< 0.001** Cohens d = 0.327	
			Timeline-Follow-Back: self-reported amount of alcohol in g (last 7 days)			IG: M = 52.91 (56.68) CG: M = 46.82 (41.18)	IG: mM = 34.3 (1.3) CG: mM = 43.7 (1.3) ***p*** **< 0.001** Cohens d = 0.419	IG: mM = 25.7 (1.5) CG: mM = 38.6 (1.4) ***p*** **< 0.001** Cohens d = 0.540	
**Zanadio** [34]						**t0 (Baseline)**	**t1 (9 months)** ^a^	**t2 (12 months)**	
	improvement of the state of health	weight reduction of at least 5%	BMI				MD = -5.67% ***p*** **= 0.001** 95% CI [-7.32, -4.02]	MD = -7.75% ***p*** **= 0.001** 95% CI: [-9.61, -5.89]	

Unless otherwise stated, all values are given as: mean/marginal mean/mean difference (standard deviation), effect size [95% confidence interval]

*CI* Confidence interval, *DiGA* Digital health application, *MCID* Minimal clinically important difference, *IG* Intervention group, *CG* Control group, *M* Mean, *mM* Marginal mean, *MD* Mean difference, *ITT* Intention-to-treat, *PP* Per protocol, *n.r.* Not reported

^a^Survey time of the primary endpoint

**Fig. 2:**
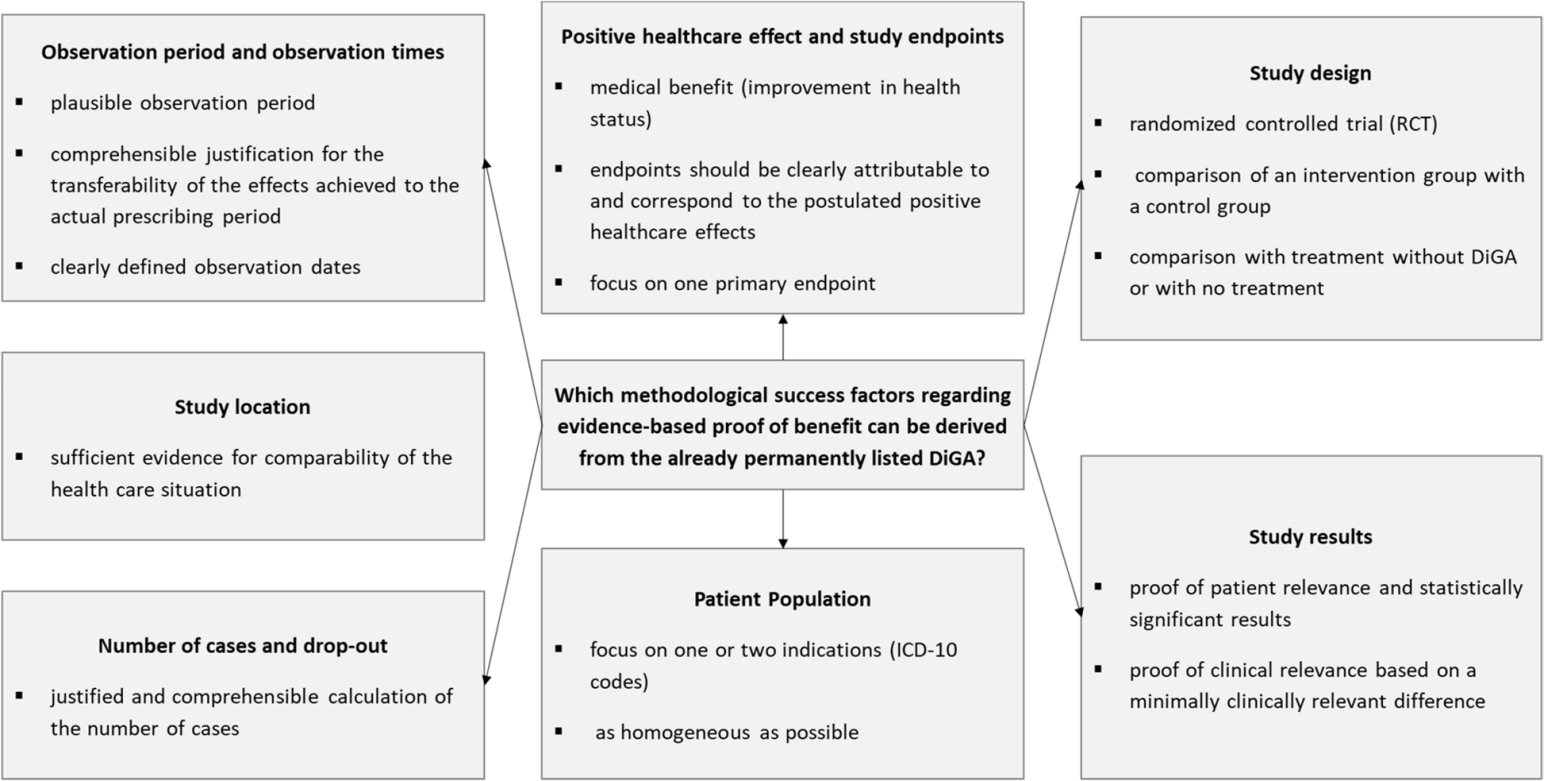
Overview of methodological success factors regarding evidence-based proof of benefit of permanently listed DiGA DiGA = Digital health application, ICD = International Classification of Diseases

After analyzing the requirements and implementation of the evidence of permanently listed DiGA, our research question, which methodological success factors regarding evidence-based proof of benefit can be derived from the already permanently listed DiGA, can be summarized with the following Fig. 2.

The requirements stated in the BfArM guideline only provide a frame of reference. The factors identified in the present analysis can help successfully implement these requirements in the standard of care.

### Further results

Although this was not a study objective, we have found the DiGA directory to lack reporting quality and transparency, which is relevant to further development of the DiGA approval process.

First, of the four DiGA initially demonstrating positive healthcare effects through systematic data evaluation, hardly any pilot study results were available. Only the Selfapy applications provisional results could be found in the DiGA directory.

Second, the DiGA directory was not sufficient for a complete analysis of the seven identified categories and the evidence produced; further sources, such as study registries, study reports and publications, had to be obtained. Finally, we found one case in which the results reported in the DiGA directory did not match to those from the study report.

## Discussion

The aim of this study was to identify BfArMs requirements of studies that prove a postulated positive healthcare effect, and, to assess the evidence of applications permanently listed in the DiGA directory.

Permanently listed DiGA meet higher requirements for demonstrating a positive healthcare effect, than those specified in the guideline. Most DiGA focused on one or two indications (ICD-10 codes), and proved a medical benefit through a RCT. All DiGA studies were able to prove patient relevance and achieved statistically significant results, however, observation periods, sample sizes and drop-out rates differed substantially among studies.

### Patient population and patient characteristics

Permanently listed mental health DiGA are most frequently represented in the DiGA directory. A focus on one or two indications (ICD-10 codes) seems to predict success for a permanent listing. This could be explained by specific and homogeneous patient populations being needed to provide adequate evidence. Surprising is however, that the BfArM guideline clearly specifies that inclusion in the DiGA directory is only possible for three or four-digit ICD-10 codes. Nevertheless, three manufacturers (Hello Better Panik, Velibra, Zanadio) achieved permanent listing using only a five-digit ICD-10 code.

In all studies aiming to demonstrate a positive healthcare effect, the proportion of women represented in the populations (in both IG and CG) was higher than that of men. A report from a German health insurance company also showed that more women use DiGA than men. Since gender-specific disease prevalence among insured patients in the (as of yet) main DiGA indications can sometimes differ greatly, this may be a result [53].

In almost all studies, the mean age of the participants was between 35 and 55 years. An analysis by a German health insurer of the age structure of all insured adults with at least one DiGA claim as of December 31^st^, 2021 showed that 27% were aged 50–59 years, followed by 22% aged 30–39 years, and 20% aged 40–49 [53].

DiGA studies published to date show that individuals with better education and employment status participated more frequently. International studies also show that the use of mobile apps is unevenly distributed in society; a US-America study showed that people with an academic education had a higher (2.8-fold) chance of using a health app, compared to those without a high school diploma. The study also showed that higher education was associated with more frequent use of digital health services [54]. A study from the Netherlands also points to social differences in the use of health apps, and showed that people who used a health app were younger and more educated, compared to those who did not [55]. An important factor that should be considered in relation to these discussed aspects is the willingness to participate in a clinical trial. Gouveia et al. (2022) showed that the willingness of younger patients to participate was significantly higher compared to older patients. In contrast, gender, lifestyle, employment status, monthly income, or education showed no influence on willingness to participate [56]. Another point influencing participation in a clinical trial is personal treatment preference [57]. In addition to willingness, attitude, and patients’ motivation to use a DiGA and/or participate in a trial, their previous experience with digital tools and their habits to use such tools for their disease self-management needs to be considered [58, 59]. Due to the influencing factors mentioned above, and the scarce data on DiGA implementation, it is not fully understood whether DiGA address the entire defined study population, or only certain subgroups. This fact underlines the high relevance of subgroup analyses for the manufacturers.

There is a need for further research on the monitoring of the actual use of DiGA by all statutory health insured in Germany, to assess whether the study populations correspond to the actual target groups. At the same time, during the process of the DiGA approval, responsible authorities should monitor whether pilot/feasibility studies, and those needed for permanent listing, may have the potential to recruit more diverse populations, and thereby increase external validity of the trials. The inherent concern is that digitally competent and literate patient populations are recruited, and other specific populations overlooked. This may further amplify the digital divide in Germany [60]. Also Stern et al. (2022) point out that equity aspects must be taken into account to ensure that health inequalities are not reinforced, or even created in the first place [12]. As such, DiGA manufacturers should be encouraged to take additional efforts to effectively recruit all genders, as well as a variety of cultural backgrounds and different levels of digital health literacy. This would enable subgroup analyses, identify those requiring additional support for adequate DiGA usage, and ultimately support individualized DiGA recommendations, according to patient characteristics, competencies and preferences [61].

### Positive healthcare effect and study endpoints

It is striking that— although patient-relevant structural and procedural improvements show high potential for improving care, especially in terms of processes — all DiGA have provided a positive care effect via a medical benefit. Lantzsch et al. (2022) pointed out needed improvement in using outcomes for patient-relevant improvement of structure and processes regarding an improved evaluation [11]. All applications were able to demonstrate an improvement in health status. Only one DiGA additionally aimed for an improved quality of life, and a reduced disease duration, which, however, could not be achieved. DiGA that provide a medical benefit in the form of a health status improvement seem to have the greatest likelihood to be permanently listed in the DiGA directory. Focusing on one primary endpoint seems to predict success for a permanent listing; the majority of the DiGA provide evidence for one defined primary endpoint. With regards to the study endpoints and measurements used, the analysis found that multiple endpoints were used, which is not surprising, given the range of indications covered. However, the heterogeneous measurements used to assess these endpoints offer a standardization potential, which will be increasingly relevant as more DiGA for the same indications are listed. To close this gap, it is possible to use Core Outcome Sets (COS). The idea of COS, is to provide a minimum set of outcome domains and a consensual set of measurement tools to be used in every clinical study with a comparable intervention and target population. Development of new, or expansion of existing COS for the evaluation of DiGA, can improve comparability of study results [62, 63].

### Study design

Although BfArM accepts study designs with a lower level of evidence for the proof of a positive healthcare effect, all manufacturers of permanently listed DiGA conducted a study with a high level of evidence. Implementing a RCT was therefore a factor increasing the likelihood of permanent inclusion in the DiGA directory. RCTs are not only conducted in the medical sector, but increasingly also in the technology sector [64], and are therefore considered a promising study design for DiGA. However, the comparatively short innovation cycles for new technologies can be an obstacle to conducting RCTs in a DiGA context. The DiGA and its individual components are usually continuously adapted and further developed by the manufacturers, so that new versions are often already available before the evaluation of the original version is completed. One success factor here can be a continuous, learning evaluation of the continuously changing DiGA process [14]. Stern et al. (2022) cite actual or perceived risks of regulatory uncertainty, as possible reasons for deciding against non-RCT studies. It may then be more risky or costly for manufacturers to subsequently switch to a more traditional study design after an unsuccessful trial [12].

Although both inter- and intra-individual comparisons are allowed, all manufacturers chose to compare an intervention group with a control group. Different methods are proposed for this comparison. DiGA manufacturers have compared to treatment without DiGA, or to no treatment. The third option — comparison with another comparable DiGA that is already permanently listed in the DiGA directory at the time of application — will become more relevant as the DiGA directory grows. Increasing in popularity is a fourth conceivable pathway; the design of a placebo DiGA, allowing blinding to be maintained. Stern et al. (2022) emphasize the relevance of establishing best practices and methods to accurately define the comparison group [12].

### Observation period and observation times

BfArM requires a description of baseline data, data collection periods, and follow-up times, but leave manufacturers flexibility in observation periods. Despite this, choosing a plausible observation period fitting to the indication, is important. If the observation period is shortened, justifying transferability of the achieved effect(s) to the prescribing period, is advisable. Stern et al. (2022) consider planning of washout phases, in which patients do not receive any therapies prior to the start of the actual intervention [12]. In any case, the observation dates must be clearly defined. In fact, almost all studies date back several years, meaning already existing data were used to prove the positive health effect. Since May 27^th^, 2020, manufacturers can submit applications to be included in the DiGA directory. The directory was started on October 6^th^, 2020. Only three trials (Kalmeda, Vivira, Zanadio) started in 2020/2021. The study for the application Vivira led to permanent listing in the DiGA directory in only 12 weeks, with a total study duration of five months.

### Study results

According to BfArM, a positive healthcare effect is considered proven if the outcome is (clinically) relevant, patient-relevant, and statistically significant. All DiGA study results were able to prove patient relevance and achieve statistical significance, and are central to successful entry in the registry.

Although the questionnaire scores improved after the application of the DiGA, the symptoms partly remained similar. One example is Hello Better Panik; here, though participants’ scores on the PAS scale (German version: Panik- und Agoraphobieskala) improved by 6.45 (IG) and 1.83 (CG) from baseline (compared to the primary endpoint), symptoms remained moderate. Similarly, Hello Better Stress und Burnout showed improved Perceived Stress Scale scores (by 8.01 (IG) and 2.19 (CG) from baseline compared to the primary endpoint), however symptoms also remained moderate. These examples show the limitations of DiGA. Somnio, however, showed differences in the Insomnia Severity Index of 7.58 (IG) and 1.22 (CG), thus improving from moderate insomnia to subthreshold insomnia; DiGA also have the potential to greatly improve users’ health.

### Further results and recommendations

In comparing the evidence required with the evidence provided by permanently listed DiGA, it was clear that transparency is not yet fully apparent. Particularly, gaining a fully comprehensive picture on some aspects (patient population, study endpoints, study location, drop-out rates, and study results) was not possible, using the DiGA directory alone. Other sources had to be consulted, such as study registers, study reports and publications. In one case, the results reported in the DiGA directory did not match those in the study report. The claimed user-friendliness of the directory must also be questioned. The user only gets an insight into the mean values/mean value comparisons. An interpretation aid for the meaning of these figures is missing.

In the future, the DiGA directory should be an essential tool for medical professionals. An important step for the spread of health apps and their integration into clinical practice, is the education of clinicians regarding available technologies [4]. A study with 51 physicians showed that half of the respondents expect to be able to identify high-quality DiGA using the DiGA directory. This group is more likely to prescribe DiGA [65]. To increase DiGA acceptance and willingness to prescribe among physicians, a transparent, complete, and correct presentation of the information is indispensable.

Future research needs are identified in the continuous evaluation of the fast-track process, with special attention to requirements of DiGA manufacturers. As an innovative and learning system in healthcare which is constantly being developed and improved (with increasingly specific requirements for DiGA manufacturers, and the growing DiGA directory), an accompanying monitoring could be a central success factor.

### Limitations

The current study combined multiple methods to analyze the available evidence of listed DiGA. However, only available studies, data, and information on the applications permanently listed in the DiGA directory were included in the analysis, due to the lack of evidence for provisionally listed applications. Therefore, recommendations may not be transferable to DiGA manufacturers aiming for provisional listing. Furthermore, evidence available for permanently listed DiGA was not identified through a systematic literature search. To limit the risk of overlooking relevant studies, numerous independent sources were used to identify DiGA-specific information. In addition to the DiGA directory, entries in study registries, study protocols, study reports, submitted publications, and manufacturers' websites, were used as information sources.

## Conclusion

The results of this analysis indicate that permanently listed DiGA meet higher standards than required by the guideline. Before prescribing a DiGA, its evidence should be carefully examined. The identified success factors provide healthcare practitioners with a transparent overview of the status quo of applications already tested. They can also support future manufacturers in the development and evaluation of their DiGA. With regards to the various endpoints used in the presented studies, future DiGA trials should focus on the most relevant outcomes, and strive towards comparability of results (especially among DiGA for the same indication). In addition, an analysis of DiGA-use in everyday care should become the subject of further implementation research. Overall, there is a need for accompanying monitoring; from the application development, through its testing during the evaluation study, to its use in everyday care. A growing DiGA system could be a beneficial improvement for the whole healthcare system.

## Data Availability

All data generated or analyzed during this study are included in this published article: **Table:** 

DiGA	Direct web links
Deprexis	DiGA directory: https://diga.bfarm.de/de/verzeichnis/450 Publications: Meyer et al. (2015): https://www.jmir.org/2009/2/e15/PDF Klein et al. (2016): https://www.karger.com/Article/Pdf/445355
Elevida	DiGA directory: https://diga.bfarm.de/de/verzeichnis/419 Publication: Pöttgen et al. (2018): https://www.researchgate.net/publication/323820297_Randomised_controlled_trial_of_a_self-guided_online_fatigue_intervention_in_multiple_sclerosis Study report: Mayer et al. (2020): https://elevida.de/downloads/studienbericht_elevida_poettgen_2018.pdf
Hello Better Diabetes and Depression	DiGA directory: https://diga.bfarm.de/de/verzeichnis/1376 Publication: Nobis et al. (2015): https://diabetesjournals.org/care/article/38/5/776/37466/Efficacy-of-a-Web-Based-Intervention-With-Mobile Study report: Balzus et al. (2021): https://hellobetter.de/wp-content/uploads/2021/08/Studienbericht_Diabetes.pdf Study protocol: Nobis et al. (2013): https://bmcpsychiatry.biomedcentral.com/articles/10.1186/1471-244X-13-306
Hello Better Panik	DiGA directory: https://diga.bfarm.de/de/verzeichnis/1513 Publication: Ebenfeld et al. (2021): https://www.jmir.org/2021/3/e20829/PDF Study report: Feiler et al. (2021): https://docplayer.org/228425224-Bezeichnung-der-klinischen-studie-smartphonebasiertes-online-training-zur-bewaeltigung-von-panikattacken-und-agoraphobie.html Study protocol: Ebenfeld et al. (2014): https://trialsjournal.biomedcentral.com/articles/10.1186/1745-6215-15-427
Hello Better Stress and Burnout	DiGA directory: https://diga.bfarm.de/de/verzeichnis/965 Publication: Heber et al. (2016): https://www.jmir.org/2016/1/e21/PDF Study report: Feiler et al. (2021): https://hellobetter.de/wp-content/uploads/2021/06/Studienbericht_HelloBetter_Stress_und_Burnout.pdf Study protocol: Heber et al. (2013): https://bmcpublichealth.biomedcentral.com/articles/10.1186/1471-2458-13-655
Hello Better Vaginismus Plus	DiGA directory: https://diga.bfarm.de/de/verzeichnis/1497 Publication: Zarski et al. (2021): https://www.researchgate.net/publication/356625888_Efficacy_of_internet-based_treatment_for_genito-pelvic_painpenetration_disorder_Results_of_a_randomized_controlled_trial Study report: Feiler et al. (2021): https://hellobetter.de/wp-content/uploads/2021/10/Studienbericht-Vaginismus-Plus.pdf Study protocol: Zarski et al. (2018): https://www.frontiersin.org/articles/10.3389/fpsyt.2017.00260/full
Kalmeda	DiGA directory: https://diga.bfarm.de/de/verzeichnis/350 Study report: Stover et al. (2022): https://drks.de/search/de/trial/DRKS00022973
Selfapy Depression	DiGA directory: https://diga.bfarm.de/de/verzeichnis/876 Publication: Krämer et al. (2022): https://formative.jmir.org/2022/4/e34330/PDF
Somnio	DiGA directory: https://diga.bfarm.de/de/verzeichnis/508 Publication: Lorenz et al. (2019): https://www.zora.uzh.ch/id/eprint/157067/1/randomized_controlled_trial_to_test_the_efficacy_of_an_unguided_online_intervention_with_automated_feedback_for_the_treatment_of_insomnia.pdf
Velibra	DiGA directory: https://diga.bfarm.de/de/verzeichnis/316 Publication: Berger et al. (2016): https://boris.unibe.ch/94877/1/effects_of_a_transdiagnostic_unguided_internet_intervention_velibra_for_anxiety_disorders_in_primary_care_results_of_a_randomized_controlled_trial.pdf Study report: Mayer et al. (2020): https://de.velibra.com/downloads/Studienbericht_velibra_Berger_2017_inkl_Append_2020-06-26.pdf
Vivira	DiGA directory: https://diga.bfarm.de/de/verzeichnis/387
Vorvida	DiGA directory: https://diga.bfarm.de/de/verzeichnis/868 Publication: Zill et al. (2019): https://www.aerzteblatt.de/archiv/205622/Wirksamkeit-einer-Internetintervention-zur-Reduktion-von-Alkoholkonsum-bei-Erwachsenen Study report: Mayer et al. (2020): https://de.vorvida.com/downloads/Studienbericht_Zill_2019_vorvida_20201105.pdf Study protocol: Zill et al. (2016): https://bmcpsychiatry.biomedcentral.com/articles/10.1186/s12888-016-0725-9
Zanadio	DiGA directory: https://diga.bfarm.de/de/verzeichnis/294 DiGA directory: https://diga.bfarm.de/de/verzeichnis/450 Publications: Meyer et al. (2015): https://www.jmir.org/2009/2/e15/PDF Klein et al. (2016): https://www.karger.com/Article/Pdf/445355 DiGA directory: https://diga.bfarm.de/de/verzeichnis/419 Publication: Pöttgen et al. (2018): https://www.researchgate.net/publication/323820297_Randomised_controlled_trial_of_a_self-guided_online_fatigue_intervention_in_multiple_sclerosis Study report: Mayer et al. (2020): https://elevida.de/downloads/studienbericht_elevida_poettgen_2018.pdf DiGA directory: https://diga.bfarm.de/de/verzeichnis/1376 Publication: Nobis et al. (2015): https://diabetesjournals.org/care/article/38/5/776/37466/Efficacy-of-a-Web-Based-Intervention-With-Mobile Study report: Balzus et al. (2021): https://hellobetter.de/wp-content/uploads/2021/08/Studienbericht_Diabetes.pdf Study protocol: Nobis et al. (2013): https://bmcpsychiatry.biomedcentral.com/articles/10.1186/1471-244X-13-306 DiGA directory: https://diga.bfarm.de/de/verzeichnis/1513 Publication: Ebenfeld et al. (2021): https://www.jmir.org/2021/3/e20829/PDF Study report: Feiler et al. (2021): https://docplayer.org/228425224-Bezeichnung-der-klinischen-studie-smartphonebasiertes-online-training-zur-bewaeltigung-von-panikattacken-und-agoraphobie.html Study protocol: Ebenfeld et al. (2014): https://trialsjournal.biomedcentral.com/articles/10.1186/1745-6215-15-427 DiGA directory: https://diga.bfarm.de/de/verzeichnis/965 Publication: Heber et al. (2016): https://www.jmir.org/2016/1/e21/PDF Study report: Feiler et al. (2021): https://hellobetter.de/wp-content/uploads/2021/06/Studienbericht_HelloBetter_Stress_und_Burnout.pdf Study protocol: Heber et al. (2013): https://bmcpublichealth.biomedcentral.com/articles/10.1186/1471-2458-13-655 DiGA directory: https://diga.bfarm.de/de/verzeichnis/1497 Publication: Zarski et al. (2021): https://www.researchgate.net/publication/356625888_Efficacy_of_internet-based_treatment_for_genito-pelvic_painpenetration_disorder_Results_of_a_randomized_controlled_trial Study report: Feiler et al. (2021): https://hellobetter.de/wp-content/uploads/2021/10/Studienbericht-Vaginismus-Plus.pdf Study protocol: Zarski et al. (2018): https://www.frontiersin.org/articles/10.3389/fpsyt.2017.00260/full DiGA directory: https://diga.bfarm.de/de/verzeichnis/350 Study report: Stover et al. (2022): https://drks.de/search/de/trial/DRKS00022973 DiGA directory: https://diga.bfarm.de/de/verzeichnis/876 Publication: Krämer et al. (2022): https://formative.jmir.org/2022/4/e34330/PDF DiGA directory: https://diga.bfarm.de/de/verzeichnis/508 Publication: Lorenz et al. (2019): https://www.zora.uzh.ch/id/eprint/157067/1/randomized_controlled_trial_to_test_the_efficacy_of_an_unguided_online_intervention_with_automated_feedback_for_the_treatment_of_insomnia.pdf DiGA directory: https://diga.bfarm.de/de/verzeichnis/316 Publication: Berger et al. (2016): https://boris.unibe.ch/94877/1/effects_of_a_transdiagnostic_unguided_internet_intervention_velibra_for_anxiety_disorders_in_primary_care_results_of_a_randomized_controlled_trial.pdf Study report: Mayer et al. (2020): https://de.velibra.com/downloads/Studienbericht_velibra_Berger_2017_inkl_Append_2020-06-26.pdf DiGA directory: https://diga.bfarm.de/de/verzeichnis/387 DiGA directory: https://diga.bfarm.de/de/verzeichnis/868 Publication: Zill et al. (2019): https://www.aerzteblatt.de/archiv/205622/Wirksamkeit-einer-Internetintervention-zur-Reduktion-von-Alkoholkonsum-bei-Erwachsenen Study report: Mayer et al. (2020): https://de.vorvida.com/downloads/Studienbericht_Zill_2019_vorvida_20201105.pdf Study protocol: Zill et al. (2016): https://bmcpsychiatry.biomedcentral.com/articles/10.1186/s12888-016-0725-9 DiGA directory: https://diga.bfarm.de/de/verzeichnis/294

## Abbreviations

BfArM: Bundesinstitut für Arzneimittel- und Medizinprodukte
CG: Control group
COS: Core Outcome Set
DiGA: Digital health application
DRKS: Deutsches Register klinischer Studien
ICD-10: International Classification of Diseases
IG: Intervention group
MDR: Medical Device Regulation
RCT: Randomized controlled trial
